# Sphingolipid Degradation in *Leishmania (Leishmania) amazonensis*

**DOI:** 10.1371/journal.pntd.0001944

**Published:** 2012-12-20

**Authors:** Agiesh Balakrishna Pillai, Wei Xu, Ou Zhang, Kai Zhang

**Affiliations:** Department of Biological Sciences, Texas Tech University, Lubbock, Texas, United States of America; Yale School of Public Health, United States of America

## Abstract

**Background:**

Human leishmaniasis is caused by more than 20 *Leishmania* species and has a wide range of symptoms. Our recent studies have demonstrated the essential role of sphingolipid degradation in the virulence of *Leishmania (Leishmania) major*, a species responsible for localized cutaneous leishmaniasis in the Old World. In this study, we investigated the function of sphingolipid degradation in *Leishmania (Leishmania) amazonensis*, an etiological agent of localized and diffuse cutaneous leishmaniasis in South America.

**Methodology/Principal Findings:**

First, we identified the enzyme LaISCL which is responsible for sphingolipid degradation in *L. amazonensis*. Primarily localized in the mitochondrion, LaISCL shows increased expression as promastigotes progress from replicative log phase to non-replicative stationary phase. To study its function, null mutants of LaISCL (*Laiscl^−^*) were generated by targeted gene deletion and complemented through episomal gene add-back. In culture, loss of LaISCL leads to hypersensitivity to acidic pH and poor survival in murine macrophages. In animals, *Laiscl^−^* mutants exhibit severely attenuated virulence towards C57BL6 mice but are fully infective towards BALB/c mice. This is drastically different from wild type *L. amazonensis* which cause severe pathology in both BALB/c and C57BL 6 mice.

**Conclusions/Significance:**

A single enzyme LaISCL is responsible for the turnover of sphingolipids in *L. amazonensis*. LaISCL exhibits similar expression profile and biochemical property as its ortholog in *L. major*. Deletion of LaISCL reduces the virulence of *L. amazonensis* and the outcome of *Laiscl^−^*-infection is highly dependent on the host's genetic background. Therefore, compared to *L. major*, the role of sphingolipid degradation in virulence is substantially different in *L. amazonensis*. Future studies may reveal whether sphingolipid degradation is required for *L. amazonensis* to cause diffuse cutaneous infections in humans.

## Author Summary

*Leishmania* parasites infect 10–12 million people worldwide, causing a spectrum of serious diseases. Among the species that infect human, *Leishmania major* is responsible for localized cutaneous disease in the Old World whereas *Leishmania amazonensis* is associated with both localized and diffuse cutaneous diseases in the Amazon region. For *L. major*, sphingolipid degradation is crucial for parasite proliferation and disease progression in mouse models. In this study, we investigated whether the function of sphingolipid degradation is conserved in *L. amazonensis*. Similar to *L. major*, *L. amazonensis* possesses a single enzyme (LaISCL) which is responsible for the turnover of sphingolipids; LaISCL is mainly associated with the mitochondrion and preferentially expressed in the infective forms of *L. amazonensis*; and deletion of LaISCL leads to poor survival under acidic conditions. While wild type *L. amazonensis* parasites are pathogenic towards all common lab mouse strains, LaISCL-null mutants show significantly lower virulence towards C57BL6 mice but are fully infective towards BALB/c mice. Therefore, although the biochemistry of sphingolipid degradation is largely conserved in *L. amazonensis*, this pathway can have drastically different effects on parasite proliferation and disease development in the mammalian host.

## Introduction

Protozoan parasites of the genus *Leishmania* are vector-borne pathogens which infect macrophages, neutrophils, and dendritic cells of mammals [1], [2]. Human infection is caused by more than 20 *Leishmania* species categorized in 2 subgenera (*Leishmania Leishmania* and *Leishmania Viannia*) and 5 complexes (*L. donovani*, *L. mexicana*, *L. tropica*, *L. hertigi*, and *L. braziliensis*) [3]. Depending on parasite species and host immune status, *Leishmania* infection can cause a wide range of symptoms including localized cutaneous lesions, diffuse cutaneous lesions, destruction of mucocutaneous membranes, and visceral diseases of the hematopoietic organs. Current drugs are plagued with low efficacy, high cost, and significant toxicity [4], [5]. A better understanding of *Leishmania*-host interaction may facilitate the development of new cost-effective treatments.

During their life cycle, *Leishmania* parasites alternate between flagellated promastigotes living in the midgut of sandflies and non-flagellated amastigotes residing in mammalian phagocytes [6]. Our recent studies of an *iscl^−^* mutant have demonstrated that sphingolipid (SL) degradation plays pivotal roles in both promastigote and amastigote stages of *Leishmania (L.) major* (commonly referred to as *Leishmania major* or *L. major*), a member of the *L. tropica* complex and one of the etiological agents for localized cutaneous leishmaniasis (LCL) in the Old World [7]. Briefly, *L. major* parasites possess a single ISCL (**I**nositol phospho**S**phingolipid phospholipase **C**-**L**ike) protein which is responsible for the degradation of both inositol phosphorylceramide (IPC, a SL synthesized by *Leishmania*) and sphingomyelin (a SL synthesized by the mammalian host) [8]. ISCL-null promastigotes (*iscl^−^*) survive poorly in culture during the stationary phase when cells are not replicative, and this defect is exacerbated by acidic pH [8], [9]. Importantly, *iscl^−^* mutants fail to cause pathology in either immunocompetent or immunodeficient mice [9]. Virulence of *iscl^−^* can be fully restored when a functional neutral sphingomyelinase (SMase) is introduced into these mutants [8], [10]. Consistent with its role as a virulence determinant, ISCL is preferentially expressed in the infective stages of *L. major*, i.e. stationary phase promastigotes and amastigotes [10]. The mechanism by which ISCL contributes to virulence is not well understood. One possibility is that the SMase activity is required for the generation of essential nutrients such as ceramide and phosphocholine (products of sphingomyelin degradation). Alternatively, ISCL may be used to remove excess sphingomyelin in the phagolysosome, which could be toxic for *L. major*.

The essentiality of ISCL in *L. major* virulence, in combination with its modest degree of homology to human neutral SMases, suggests that this enzyme possesses the potential of being a drug target. Because more than 20 species of *Leishmania* species can infect human, it is important to investigate whether the role of ISCL is conserved in these parasites. Here we extend the study of SL degradation to *Leishmania (L.) amazonensis* (commonly referred to as *Leishmania amazonensis* or *L. amazonensis*), which belongs to the *L. mexicana* complex and mostly found in South America [11], [12]. In addition to differences in geographic distribution and insect vector preference, *L. amazonensis* infection is distinct from *L. major* infection in clinical manifestation and host immune response. While *L. major* mainly causes LCL, *L. amazonensis* is associated with a range of symptoms in humans from LCL to diffuse cutaneous leishmaniasis (DCL) [13]. DCL is a rare, chronic form of leishmaniasis in which the initial cutaneous lesion is followed by the formation of secondary or satellite lesions all over the body [14]. Compared to LCL, DCL is more resistant to conventional therapy [15]. In human infections, *L. amazonensis*-induced DCL is characterized by high lesional macrophage-to-T cell ratio, uncontrolled parasite proliferation, and a lack of delayed hypersensitivity reaction, indicating that the cell-mediated immune mechanism is incapable of limiting the leishmanial infection (a “hyposensitivity” phenotype) [16] [12].

In murine models of cutaneous leishmaniasis, *L. major* infection induces polarized T cell response which dictates disease outcome, *e.g.* BALB/c mice are susceptible to *L. major* due to a Th2-dominated response leading to uncontrolled parasite growth and severe pathology, whereas C57BL6 mice are resistant due to a protective Th1-dominated response [17]. In contrast, *L. amazonensis* causes non-healing lesions in almost all inbred lab strains of mice in the absence of a Th2-dominance [18] [19]. A low level of mixed Th1/Th2 response has been observed in *L. amazonensis*-infected hosts [18], [20], [21]. In macrophages, *L. amazonensis* and other members of the *L. mexicana* complex are capable of forming large parasitophorous vacuoles (PV) with heavy parasite loads [22]. Such communal PVs (not formed by *L. major*) continuously undergo fusion with lysosomes and may protect *L. amazonensis* amastigotes by diluting the leishmaniacidal effects of nitric oxide (NO) and reactive oxygen species (ROS) from the host [23], [24].

To investigate the function of SL degradation in *L. amazonensis*, we generated a LaISCL-null mutant equivalent to the *iscl^−^* mutant in *L. major*. Our results show that while the biochemistry of SL degradation is largely conserved in *L. amazonensis*, its role in parasite proliferation and disease development depends on the genetic background of the mammalian host. This study expands our understanding of SL metabolism and provides new information into the complex nature of *Leishmania* pathogenesis.

## Methods

### Materials

N-[6-[(7-nitro-2-1,3-benzoxadiazol-4-yl)amino]hexanoyl]-sphingosine-1-phosphocholine (NBD C6-sphingomyelin) and MitoTracker Red 580 was purchased from Life Technologies (Grand Island, NY). N-[12-[(7-nitro-2-1,3-benzoxadiazol-4-yl)amino]dodecanoyl]-sphingosine-1-phosphoinositol (NBD C12-IPC) was custom-synthesized by Avanti Polar lipids (Alabaster, AL). The rabbit anti-*L. major* ISCL peptide antibody was custom-produced by the Open Biosystems, Inc (Huntsville, AL). ELISA kits to measure IFN-γ, IL-4, and IL-10 production were purchased from eBioscience Inc (San Diego, CA). All other reagents were purchased from Sigma-Aldrich (St. Louis, MO) or Fisher Scientific (Pittsburgh, PA) unless specified otherwise.

### Molecular constructs

The *L. amazonensis ISCL* (*LaISCL*) open reading frame was amplified from *L. amazonensis* genomic DNA by PCR using primers #141/#58 which were synthesized according to the sequence of *L. mexicana ISCL* (TryTrypDB LmxM.08.0200). The resulting 1.9-Kb fragment was cloned in pXG (a high copy expression vector in *Leishmania*) [25] to generate pXG-*LaISCL* (strain B163). To generate knockout constructs, the predicted 5′-and 3′-flanking regions of *LaISCL* were PCR amplified using primers #161/#143 and #144/#145, respectively. The resulting DNA fragments were cloned in tandem in the pUC18 vector. Genes conferring resistance to puromycin (*PAC*) and blasticidin (*BSD*) were then inserted between the 5′- and 3′- flanking regions to generate pUC-KO-*LaISCL::PAC* (strain B178) and pUC-KO-*LaISCL::BSD* (strain B177). Primers used in this study were summarized in Table S1. All constructs were confirmed by restriction enzyme digestion and DNA sequencing.

### *Leishmania* culture and genetic manipulation

*L. amazonensis* (MHOM/BR/77/LTB0016) promastigotes were cultured at 26°C in M199 medium (pH 7.4) with 10% fetal bovine serum and additional supplements [26]. Metacyclics were isolated from day 3 stationary phase promastigotes using the density centrifugation method [27]. *L. amazonensis* axenic amastigotes were cultured at 33°C in Grace's insect cell culture medium (the pH was adjusted to 5.3) with L-glutamine and 20% fetal bovine serum [28], [29]. Purification of lesion amastigotes were performed as previously described [10].

To generate *Laiscl^−^* mutants (*▵LaISCL::PAC/▵LaISCL::BSD*), the *LaISCL* alleles from wild type *L. amazonensis* parasites (*La* WT) were sequentially replaced by *PAC* and *BSD* resistance genes as previously described for the generation of *L. major iscl^−^* mutants [8]. To confirm the deletion of *LaISCL*, genomic DNA was digested with *Spe*I, resolved on a 0.7% agarose gel, transferred to a nitrocellulose membrane, and hybridized with a [^32^P]-labeled DNA probe corresponding to a 550-bp downstream flanking region of *LaISCL*. These null mutants were maintained in 10 µg/ml of puromycin and 10 µg/ml of blasticidin. To complement these mutants, pXG-*LaISCL* was transfected into *Laiscl^−^* to generate the episomal add-back, referred to as *Laiscl^−^/+LaISCL*. These add-back parasites were grown in 20 µg/ml of G418. To prevent virulence loss during in vitro culture and genetic manipulation, stationary phase promastigotes of *La* WT, *Laiscl^−^* and *Laiscl^−^/+LaISCL* were passed through BALB/c mice at ∼2×10^7^ cells/mouse and recovered one month later as previously described [30]. These parasites were then converted back to promastigotes and used in mouse footpad infection and macrophage infection.

### Measuring cell growth and pH tolerance

Promastigotes or axenic amastigotes were inoculated in appropriate media (starting density for promastigotes: 1.0×10^5^ cells/ml; for axenic amastigotes: 1.0×10^6^ cells/ml). Growth rates were determined by counting culture density at designated times with a hemacytometer. Cell viability was determined by flow cytometry after staining with propidium iodide as previously described [31]. Percentages of round cells (defined as those promastigotes with the long axis shorter than twice the length of the short axis) were determined by microscopy as previously described [8]. To determine the sensitivity of *L. amazonensis* parasites to acidic pH, promastigotes were cultured in an acidic medium (same as the regular M199 medium except the pH was adjusted to 5.0 with hydrochloric acid) and growth rate and cell viability were determined as described [9].

### Western blot and immunofluorescence microscopy

*L. amazonensis* promastigotes or lesion-derived amastigotes were suspended in phosphate buffered saline (PBS) at 5×10^7^ cells/ml and boiled in SDS sample buffer for 5 minutes. Western blot was performed as previously described using the rabbit anti-*L. major* ISCL peptide antibody [10]. Results were quantified using a FluoroChem E imager (Protein Simple).

Immunofluorescence microscopy of *L. amazonensis* promastigotes or lesion-derived amastigotes was performed as previously described [10]. Briefly, formaldehyde fixed parasites were attached to poly-lysine coated cover slips and permeabilized with ice-cold ethanol. Cells were labeled with the rabbit anti- *L. major* ISCL antibody (1∶500 in 2% bovine serum albumin prepared in PBS) for 30 minutes, and then incubated with a goat anti-rabbit IgG-FITC (1∶1000 dilution) for 30 minutes. 350 nM of Mitotracker Red 580 (Molecular Probes/Life Technologies) was then applied for 30 minutes, followed by staining with 2.5 µg/ml of Hoechst 33342 for 10 minutes. Images were acquired using an Olympus BX51 Upright Fluorescence Microscope equipped with a digital camera.

### Assays for SL degradation

Log phase *L. amazonensis* promastigotes (1–8×10^6^ cells/ml) were suspended in a lysis buffer (25 mM Tris pH 7.5, 0.1% Triton X100, 1× protease inhibitor) at 2.0×10^8^ cells/ml and incubated for 5 min on ice. Protein concentration was determined using a micro-BCA kit (Pierce). The neutral SMase assay and IPCase assay were performed as previously described [8]. Each reaction contained 40 µg of *L. amazonensis* protein and 0.8 nmol of NBD C6-sphingomyelin or 0.8 nmol of NBD C12-IPC. 0.1 unit of *Bacillus cereus* SMase or phosphatidylinositol phospholipase C was used as a positive control and boiled WT lysate was used as a negative control. Activities were quantified using a Storm 860 phosphoimager and converted to pmol/(µg×hour) after subtracting the value of negative control.

### Phospholipid analysis by mass spectrometry

Total lipids from stationary phase promastigotes were extracted using the Bligh-Dyer method and analyzed by electrospray ionization mass spectrometry (the negative ion mode) as previously described [31].

### Ethics statement for mouse use

The use of mice in this study was approved by the Animal Care and Use Committee at Texas Tech University (PHS Approved Animal Welfare Assurance NO. A3629-01). C57BL6 mice (female, 7–8 weeks old) and BALB/c mice (female, 7–8 weeks old) were purchased from Charles River Laboratories International (Wilmington, MA). Mice were housed and cared for in the facility operated by the Animal Care and Resources Center at Texas Tech University adhering to the institution's guidelines for animal husbandry. The facility was inspected monthly and animals were monitored daily by staff members. A complete range of clinical veterinary services was available on a 24-hour basis and includes consultation, diagnostic work-up and clinical care. Lab personnel are trained to use proper restraining and injection techniques to reduce pain and distress of animals.

Mice were under anesthesia (through the peritoneal injection of ketamine hydrochloride/xylazine) during recurring procedures including the injection of *Leishmania* parasites into footpads, the recovery of parasites from infected mice, and the measurement of lesion size using a caliper. Usually, no more than one procedure was performed on one mouse within a week. To prevent any potential secondary infections and to reduce any potential pain/distress, mice were monitored carefully (twice a week for appearance, size, movement, and general health condition) and euthanized when the lesions became too large (>2.5 mm for footpad infection). For the isolation of femur cells, draining lymph nodes (dLNs), and the determination of parasite numbers in the infected footpads, mice were euthanized by CO_2_ asphyxiation prior to operations.

### Macrophage infection and mouse footpad infection

Bone marrow-derived macrophages were generated from the femur of BALB/c mice [10]. Macrophage infection was performed using day 3 stationary phase *L. amazonensis* promastigotes at a ratio of five parasites per macrophage (multiplicity of infection = 5∶1) as previously described [32].

Footpad infections of BALB/c mice and C57BL6 mice were performed as previously described [33], [34] using day 3stationary phase promastigotes (1×10^6^ cells/mouse) or lesion-derived amastigotes (1×10^4^ cells/mouse). Six mice were used in each group. Parasite numbers in the infected footpad were determined by limiting dilution assay [34].

### Quantitation of cytokine production from lymphocytes

To prepare lymphocyte suspension, *L. amazonensis*-infected mice (two from each group) were sacrificed and dLNs were collected. To measure cytokine production, lymphocytes from dLNs were cultured in 24-well plates (4×10^6^ cells/ml) and stimulated with soluble *L. amazonensis* antigen (SLA) (equivalent to 8×10^6^ parasites/ml; generated by repeated freeze-thaw cycles) for 72 hours. Supernatants were assayed for IFN-γ, IL-4, or IL-10 using appropriate ELISA kits [35]. To offset potential variations in dLN cell numbers among wells, the ratio of SLA-stimulated over un-stimulated for each sample was recorded.

### Statistical analysis

The difference between two experimental groups was determined by the Student's *t* test using Sigmaplot11.0 (Systat Software Inc, San Jose, CA). *P* values indicating statistical significance were grouped into values of <0.05 and <0.01.

### Accession numbers/ID numbers for genes and proteins mentioned in this study

- *L. amazonensis* ISCL (LaISCL): GenBank JX131379
- *L. major* ISCL (LmISCL): TriTryDB LmjF.08.0200
- *L. mexicana* ISCL (LmexiISCL): TriTryDB LmxM.08.0200

## Results

### Identification and targeted deletion of LaISCL

**Figure 1:**
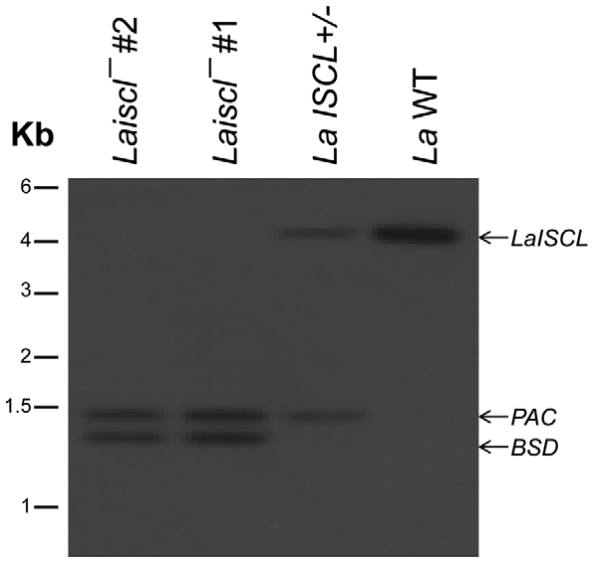
Replacement of *LaISCL* alleles by antibiotic resistance marker genes. Genomic DNAs from *La* WT, *LaISCL+/−* (*▵LaISCL::PAC/LaISCL*), and *Laiscl^−^* (*▵LaISCL::PAC/▵LaISCL::BSD*; clone #1 and #2) were digested and probed with a radioactive probe that recognized a 550-bp downstream region of *LaISCL*.

Because the genome of *L. amazonensis* is not sequenced, we first synthesized oligonucleotides based on the sequence of *L. mexicana* ISCL (TriTryDB: LmxM.08.0200). These oligonucleotides (summarized in Table S1) were then used to amplify the open reading frame and 5′-/3′-flanking regions of *LaISCL* from *L. amazonensis* genomic DNA. The open reading frame of *LaISCL* (GenBank JX131379) encodes a protein of 645 amino acids with 86% identity to *L. major* ISCL and 98% identity to *L. mexicana* ISCL (Fig. S1). Similar to *L. major* ISCL, LaISCL possesses a P-loop motif (found in phosphatases and nucleotide-binding proteins and may be essential for catalytic efficiency) [36] and two putative transmembrane helices near the C-terminus (Fig. S1). To understand the function of this protein in *L. amazonensis*, null mutants of *LaISCL* (referred to as *Laiscl^−^*) were generated through two sequential rounds of targeted gene deletion. Southern blot analysis confirmed the loss of *LaISCL* in *Laiscl^−^* (Fig. 1; *LaISCL+/^−^* represents the heterologous parasite in which one of the two *LaISCL* alleles is deleted). To complement the mutant, a high copy number episome carrying *LaISCL* (pXG-*LaISCL*) was introduced into *Laiscl^−^* and the add-back strain is referred to as *Laiscl^−^/+LaISCL*.

### Growth of *Laiscl^−^* mutants as promastigotes and axenic amastigotes

**Figure 2:**
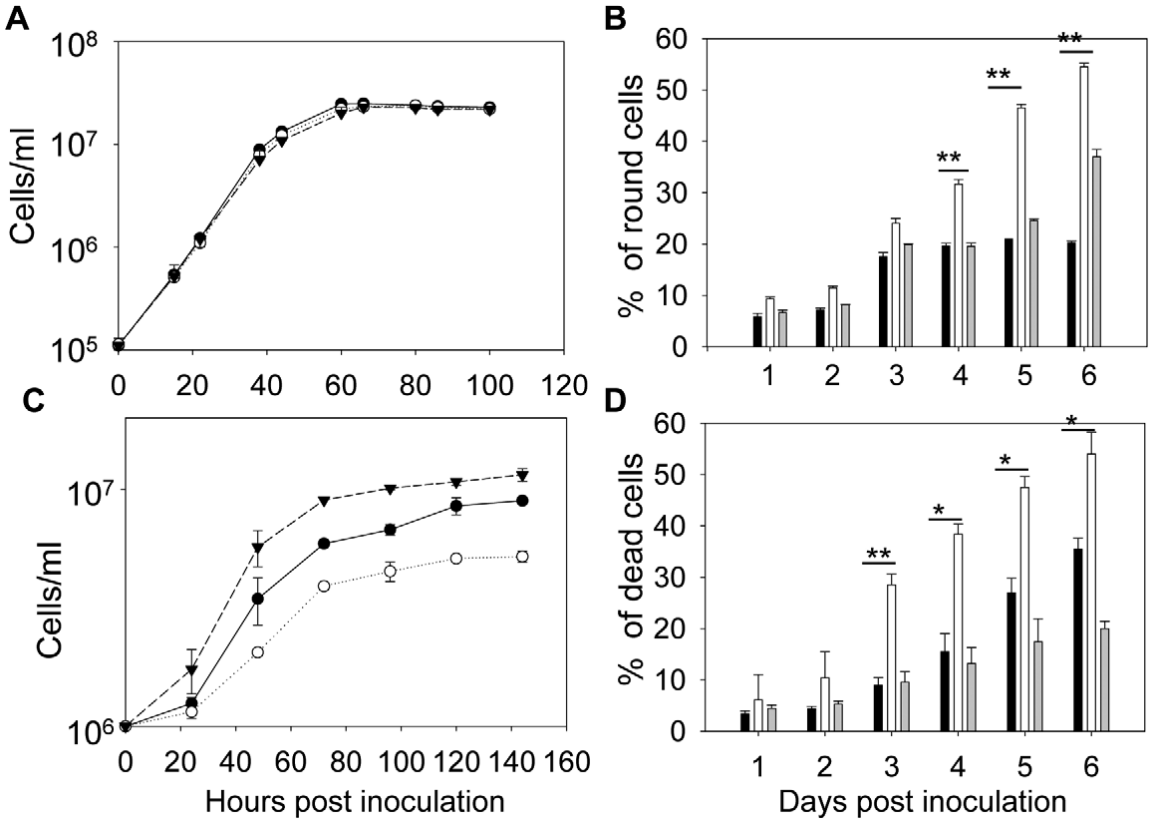
*LaISCL* is required for the maintenance of cell shape and viability in stationary phase. Promastigotes (**A–B**) or axenic amastigotes (**C–D**) were inoculated in appropriate media and culture densities were determined every 8–12 hours in **A** and **C** (•: *La* WT, ○: *Laiscl^−^*, ▾: *Laiscl^−^/+LaISCL*). Percentages of round cells in promastigotes (**B**) and percentages of dead cells in axenic amastigotes (**D**) were analyzed daily. In **B** and **D**, black bars: *La* WT, white bars: *Laiscl* ^−^, grey bars: *Laiscl* ^−^/+*LaISCL*. Error bars represent standard deviations (*: *p*<0.05, **: *p*<0.01).

In culture, *Laiscl^−^* promastigotes could proliferate from early log phase (<1×10^6^ cells/ml) to stationary phase (2.8–3.2×10^7^ cells/ml) with a doubling time of ∼7 hours (Fig. 2A and Fig. S2A). Their growth rate and maximal culture density are similar to what were observed with *L. amazonensis* wild type (*La* WT) and *Laiscl^−^/+LaISCL* parasites (Fig. 2A and Fig. S2A). After entering stationary phase (3 days in culture in Fig. 2A–B), *Laiscl^−^* promastigotes became more round in shape. Microscopic observation revealed that 46–55% of *Laiscl^−^* were round in late stationary phase whereas only 17–22% of *La* WT promastigotes showed similar morphology (Fig. 2B). Meanwhile, more dead cells were detected in *Laiscl^−^* than in *La* WT during late stationary phase (Fig. S2B), suggesting that these round cells were not healthy. We also examined whether *Laiscl^−^* promastigotes were sensitive to acidic pH in stationary phase by culturing them in an acidic medium (pH 5.0). As shown in Fig. S2C–D, *Laiscl^−^* mutants died almost completely by day 5 in stationary phase and this defect was more severe than what was observed under neutral pH (Fig. S2B), indicating that these parasites are vulnerable to acidity. It is worth mentioning that when promastigotes were cultured in neutral condition (Fig. 2A–B and Fig. S2A–B), the medium was slightly acidified in late stationary phase (∼0.5 lower than in log phase). This indicates that the death of *Laiscl^−^* may be partially attributed to their hypersensitivity to acidic pH, although other factors such as nutrient depletion and toxic waste build-up are likely involved as well. As an important control, restoration of *LaISCL* expression (*Laiscl^−^/+LaISCL* in Fig. 2 and Fig. S2) largely reversed the morphological and viability defects of *Laiscl^−^* promastigotes. Finally, despite their abnormality in culture, *Laiscl^−^* mutants still formed metacyclics in stationary phase at a similar rate as *La* WT (Fig. S3).

Next, we examined the growth and viability of *Laiscl^−^* as axenic amastigotes. Unlike *L. major*, promastigotes of *L. amazonensis* can be converted into axenic amastigotes in culture [37]. When *Laiscl^−^* promastigotes were exposed to an acidic amastigote-inducing medium at a higher temperature, within 24 hours, they lost flagella and became round (data not shown). This transition indicates that LaISCL is not required for the transformation of promastigotes into axenic amastigotes. However, as shown in Fig. 2C, the growth rate and maximal culture density of *Laiscl^−^* axenic amastigotes were lower comparing to *La* WT. In addition, starting from the third day of amastigote culture, more dead cells were detected in the mutant (29–58%) than in *La* WT (8–35%) parasites (Fig. 2D). This defect may be related to the hypersensitivity of *Laiscl^−^* to acidic pH (since the amastigote medium was maintained at pH 5.3). Finally, the add-back parasites (*Laiscl^−^/+LaISCL*) survived and grew better than *La* WT (Fig. 2C–D), suggesting that increased LaISCL expression can be beneficial for *L. amazonensis* under certain conditions such as high temperature and low pH.

### Temporal and spatial expression of LaISCL

**Figure 3:**
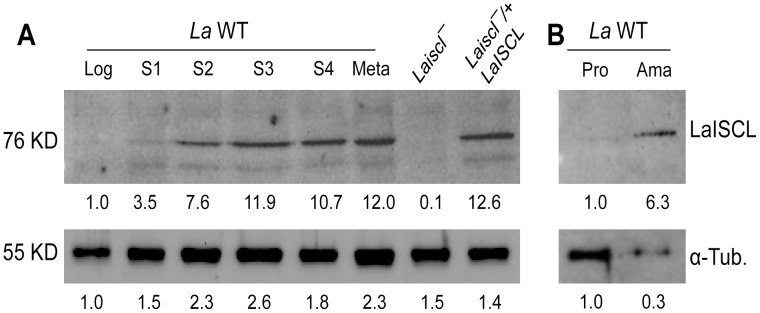
Increased expression of LaISCL in the infective stages of *L. amazonensis*. (**A**) Promastigote lysates from *La* WT (Log: log phase; S1–S4: day 1–4 in stationary phase; Meta: metacyclics), *Laiscl^−^* (log phase), and *Laiscl^−^/+LaISCL* (log phase) were analyzed by western blot using the anti-LmISCL antibody (top) or anti-α-tubulin antibody (bottom). (**B**) Immunoblot of cell lysates from log phase *La* WT promastigotes (Pro) and lesion-derived amastigotes (Ama). Relative intensities of ISCL and tubulin bands were determined using a FluoroChem E imager and shown below the blots. Each lane contained material from 5×10^5^ cells.

The abundance of LaISCL protein in *La* WT promastigotes and amastigotes was examined by Western blot, using a peptide antibody which recognizes a 16 amino acids (amino acid 241–256) epitope in *L. major* ISCL (Fig. S1). The high degree of similarity between LaISCL and *L. major* ISCL (11 out 16 amino acids within the epitope are conserved; Fig. S1) suggests that the anti-*L. major* ISCL antibody may cross react with LaISCL. Indeed, a 76 KD band was detected in the promastigote lysates of *La* WT but not *Laiscl^−^* (Fig. 3A), which was consistent with the predicted molecular weight of LaISCL. Compared to *La* WT, *Laiscl^−^/+LaISCL* parasites synthesized 8–10 times more LaISCL protein due to the high-copy number of pXG-*LaISCL* episome (Fig. 3A) [25]. The cellular level of LaISCL protein increased significantly (5–6 fold) when *La* WT promastigotes went from replicative log phase to non-replicative but infective stationary phase and metacyclic phase (Fig. 3A). In addition, *La* WT amastigotes purified from infected BALB/c mice contained much more LaISCL protein than log phase promastigotes (Fig. 3B). In summary, the stage–dependent expression of LaISCL suggests that it plays a vital role in the infective forms (i.e. late stationary phase promastigotes, metacyclics, and amastigotes) of *L. amazonensis*.

**Figure 4:**
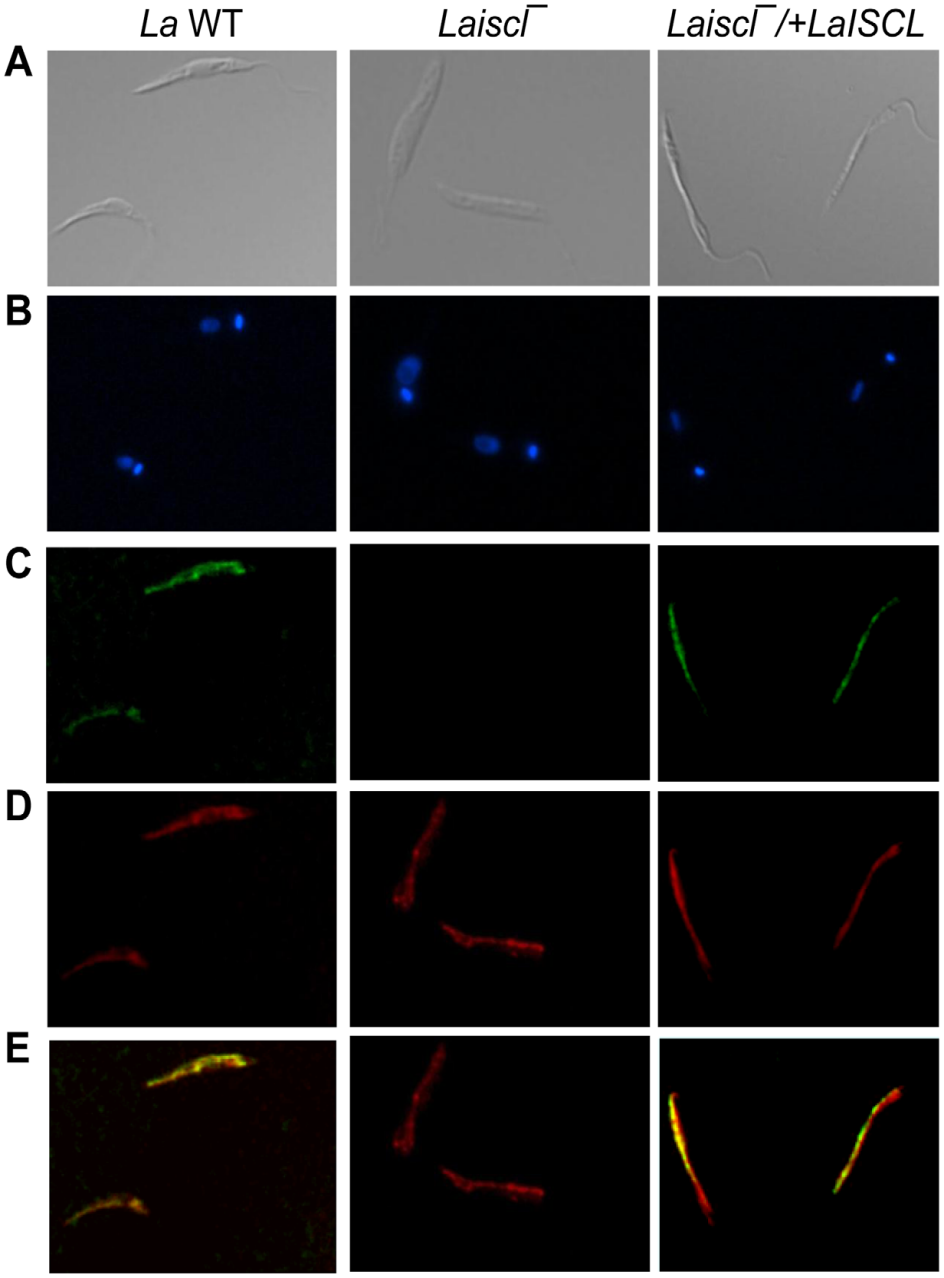
Localization of LaISCL protein in promastigotes. Day 1 stationary phase promastigotes of *La* WT (left column), *Laiscl* ^−^ (middle column), and *Laiscl* ^−^/+*LaISCL* (right column) were analyzed by immunofluorescence microscopy. (**A**): differential interference contrast images; (**B**): DNA staining using Hoechst 33242; (**C**): immuno-staining with rabbit anti-LmISCL antibody, followed by goat-anti-rabbit IgG-FITC; (**D**): labeling with Mitotracker Red 580; (**E**): merge of **C** and **D**.

To determine the localization of LaISCL, *La* WT promastigotes were labeled with the anti-*L. major* ISCL antibody for immunofluorescence microscopy. As shown in Fig. 4, a substantial overlap between LaISCL and Mitotracker Red 580 (a mitochondrial marker) was detected in *La* WT and *Laiscl^−^/+LaISCL* parasites, while LaISCL was invisible in *Laiscl^−^* as expected. In *La* WT amastigotes (isolated from infected mice), the distribution of LaISCL also resembled the pattern of mitochondrion (Fig. S4). Together, these results indicate that LaISCL is mainly localized in the mitochondria or mitochondria-associated ER membranes, which is similar to what we previously described for ISCL in *L. major* [8], [10].

### LaISCL is responsible for the SMase and IPCase activity in *L. amazonensis*

**Figure 5:**
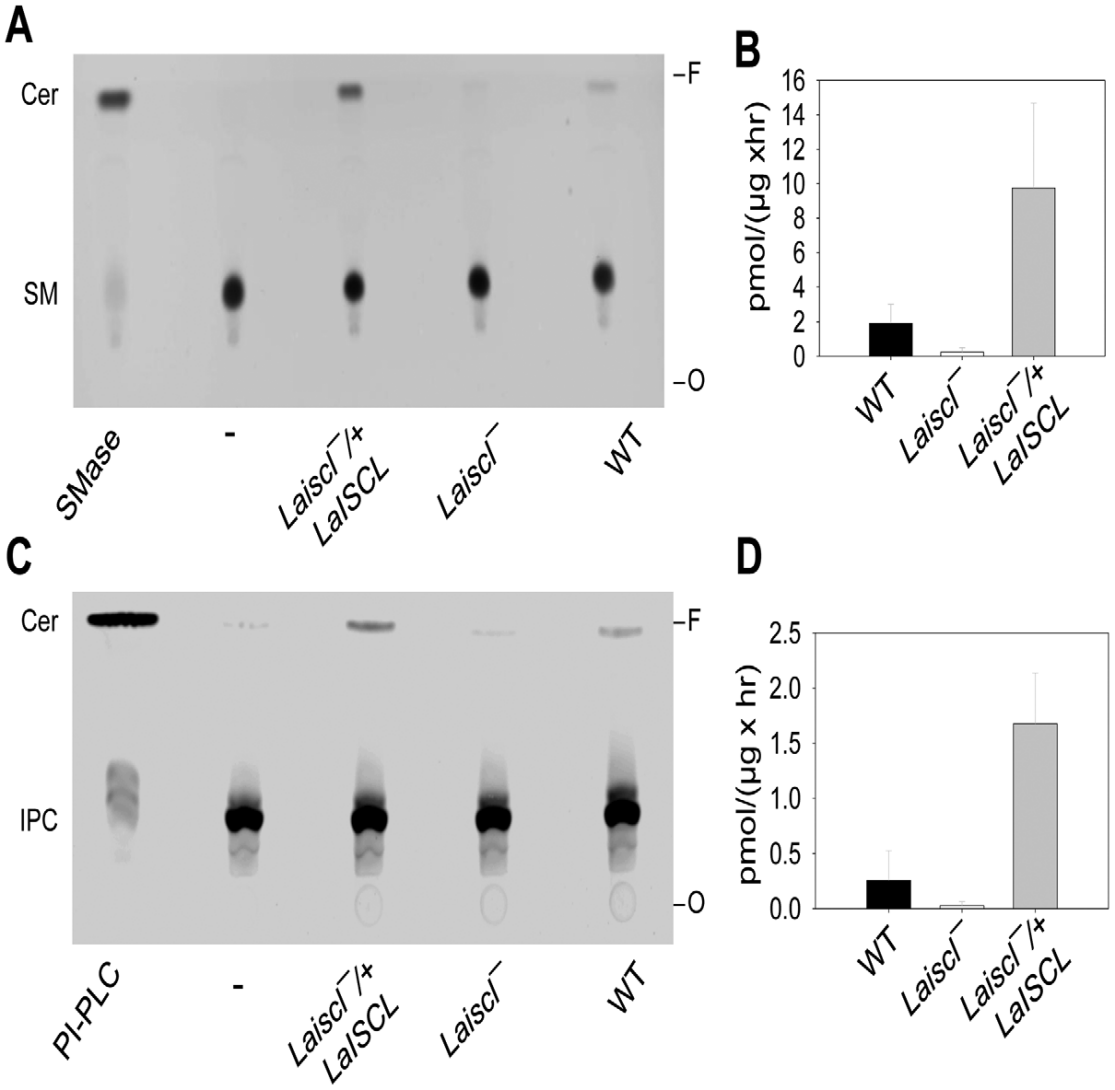
LaISCL is required for the hydrolysis of sphingomyelin and IPC in *L. amazonensis*. Promastigote lysates were incubated with TX100-based micelles containing either NBD-SM (**A–B**) or NBD-IPC (**C–D**) as described in Methods. Lipids were then extracted and separated on TLC plates (**A** and **C**). The activity of SMase (**B**) or IPCase (**D**) was calculated based on the amount of ceramide produced and the amount of protein in each sample. 0.1 unit of *B. cereus* SMase (**A**) and *B. cereus* PI-PLC (**C**) were used as positive controls. Boiled *La* WT cell lysate was used as negative controls (-).

To determine whether LaISCL is required for the neutral SMase activity, whole cell extracts from *La* WT, *Laiscl^−^* and *Laiscl^−^/+LaISCL* promastigotes were incubated with a NBD-labeled C6 sphingomyelin and lipid products were analyzed afterwards by TLC. As illustrated in Fig. 5A–B, fluorescent ceramide was generated by *La* WT parasites but not by *Laiscl^−^* parasites. In addition, a higher level of SMase activity was detected in *Laiscl^−^/+LaISCL* (4–7 times more than *La* WT) due to the overexpression of LaISCL in these add-back cells (Figs. 3 and 5A–B). Similar results were obtained when a NBD-labeled C12 IPC was used as substrate: while cell lysates from *La* WT and *Laiscl^−^/+LaISCL* exhibited IPCase activity, *Laiscl^−^* parasites failed to hydrolyze IPC into ceramide (Fig. 5C–D). Therefore, LaISCL is responsible for both the SMase and IPCase activity in *L. amazonensis*. In *La* WT and *Laiscl^−^/+LaISCL* cell lysates, the apparent SMase activity was 6–10 fold higher than IPCase (Fig. 5B and D), suggesting that sphingomyelin is the preferred substrate for LaISCL.

We also examined the phospholipid composition of *Laiscl^−^* promastigotes by mass spectrometry in the negative ion mode. Compared to *La* WT, *Laiscl^−^* parasites contained a higher level of IPC as the 778.60 peak representing a deprotonated IPC [31] was the strongest peak in *Laiscl^−^* but not *La* WT (Fig. S5A–B). Notably, the level of IPC was not fully reversed in the *Laiscl^−^/+LaISCL* parasites (Fig. S5C), which could be due to the separate localization of LaISCL (mitochondrion) and IPC (plasma membrane). Besides IPC, other phospholipid species including phosphatidylethanolamine and phosphatidylinositol did not show much difference between *La* WT and *Laiscl^−^* (Fig. S5). Therefore, deletion of LaISCL leads to accumulation of IPC but does not affect the overall lipid composition in *L. amazonensis*.

### LaISCL is required for *L. amazonensis* infection in C57BL6 mice but not in BALB/c mice

**Figure 6:**
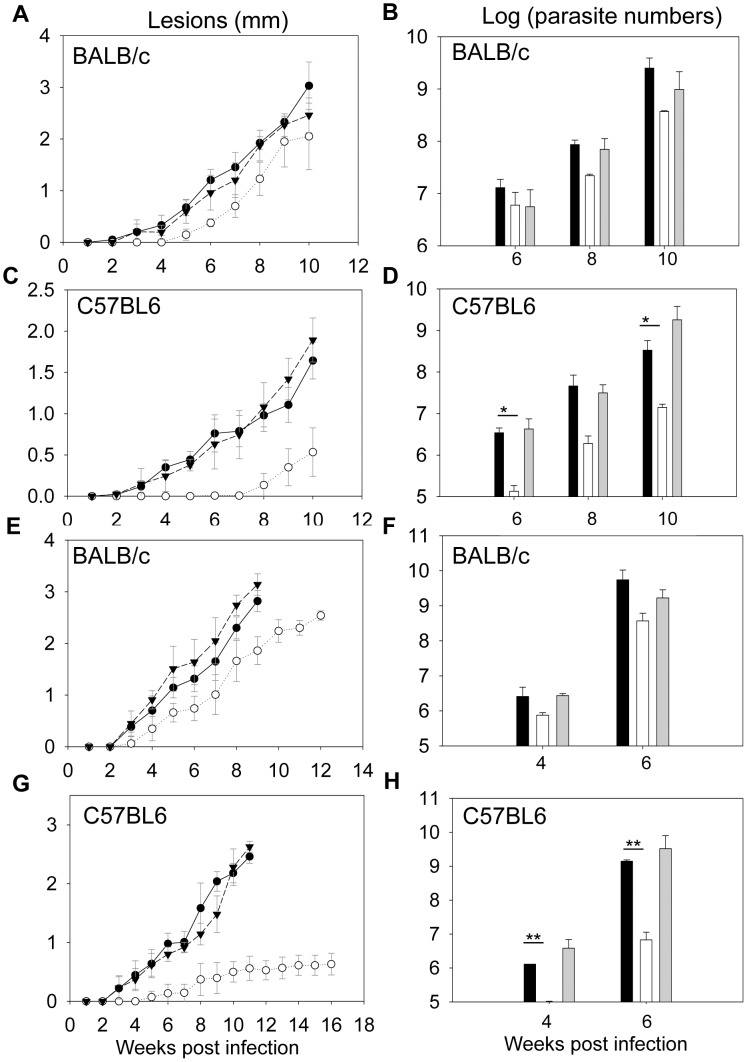
*Laiscl* ^−^ mutants are virulent in BALB/c mice but attenuated in C57BL6 mice. BALB/c mice or C57BL6 mice were infected in the footpads with stationary phase promastigotes (1×10^6^ parasites/mouse) (**A–D**) or lesion-derived amastigotes (1×10^4^ parasites/mouse) (**E–H**). Footpad lesions were recorded weekly in **A**, **C**, **E** and **G** (•: WT, ○: *Laiscl* ^−^, ▾: *Laiscl* ^−^/+*LaISCL*). Parasite numbers in the infected footpads were determined at the indicated times by limiting dilution assay and summarized in **B**, **D**, **F** and **H** (black bars: *La* WT, white bars: *Laiscl* ^−^, grey bars: *Laiscl* ^−^/+*LaISCL*). Error bars represent standard deviations (*: *p*<0.05, **: *p*<0.01).

To determine the role of LaISCL in virulence, *L. amazonensis* promastigotes were injected into the footpads of BALB/c or C57BL6 mice and the development of pathology was monitored (Fig. 6A–D). In BALB/c mice, *Laiscl^−^* mutants showed a slight delay (∼2 weeks) in lesion formation but their overall disease-inducing ability was comparable to that of *La* WT and *Laiscl^−^/+LaISCL* promastigotes (Fig. 6A). Numbers of *Laiscl^−^* parasites in the infected footpads were also similar to those of *La* WT and *Laiscl^−^/+LaISCL* parasites at 6, 8, and 10 weeks post infection (Fig. 6B), suggesting that LaISCL is not required for *L. amazonensis* infection in BALB/c mice. However, when the same experiment was performed in C57BL6 mice, *Laiscl^−^* mutants did not cause any detectable disease for the first 7 weeks (Fig. 6C). After the delay, mice infected by *Laiscl^−^* only developed very small lesions (<0.8 mm) (Fig. 6C). This is in steep contrast to the C57BL6 mice infected by *La* WT or *Laiscl^−^/+LaISCL* promastigotes, which developed noticeable pathology after 2 weeks and those lesions grew to 1.5–2.3 mm in 10 weeks (Fig. 6C). Consistent with the reduced pathology, *Laiscl^−^*-infected C57BL6 mice contained significantly less parasites than those infected with *La* WT or *Laiscl^−^/+LaISCL* (Fig. 6D). Together, these results suggest that LaISCL plays an important role in the proliferation of *L. amazonensis* and the development of pathology in C57BL6 mice.

We also examined whether LaISCL is required for the virulence of *L. amazonensis* amastigotes. To do so, amastigotes were purified from the footpads of infected BALB/c mice and then immediately injected into naïve BALB/c or C57BL6 mice. As shown in Fig. 6E–F, amastigotes of *Laiscl^−^* could proliferate and induce pathology in BALB/c mice at a similar rate as the amastigotes of *La* WT and *Laiscl^−^/+LaISCL*. In contrast, *Laiscl^−^* amastigotes were severely attenuated in C57BL6 mice (Fig. 6G–H). Overall, these amastigote infection data are highly similar to the promastigote infection results.

### *Laiscl^−^* parasites survived poorly in murine macrophages

To better understand the role of *LaISCL* in parasites-host interaction, we conducted murine macrophage infection experiments *in vitro*. Macrophages were induced from the bone marrow cells of BALB/c mice and infected with *L. amazonensis* promastigotes (Fig. S6A–B). Fractions of infected macrophages and the number of parasites in 100 macrophages were monitored at 2–72 hours post infection (Fig. S6A–B). Compared to *La* WT and *Laiscl* ^−^ */+LaISCL* parasites, *Laiscl* ^−^ mutants survived much poorly in macrophages, especially during the first 24 hours of infection (Fig. S6A–B). Similar results were obtained using bone marrow macrophages from C57BL6 mice (Fig. S6C–D). Therefore, although *Laiscl* ^−^ mutants are fully virulent against BALB/c mice, they are compromised in macrophage infection.

### Cytokine response in *Laiscl^−^* infected mice

**Figure 7:**
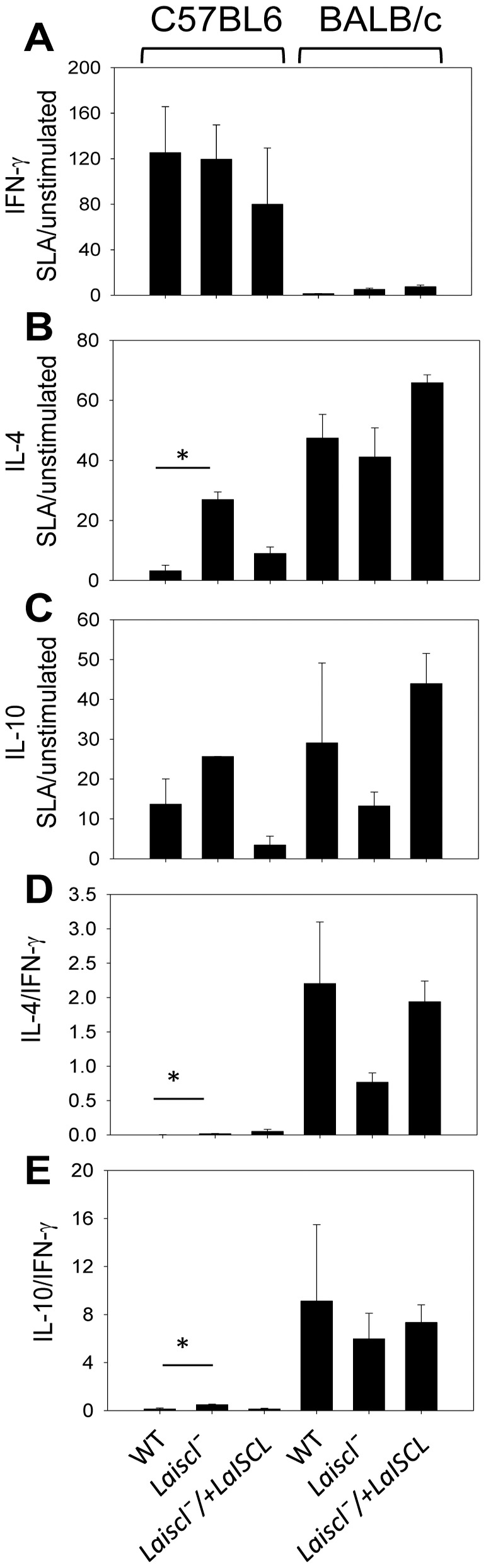
Cytokine production in *Laiscl* ^−^-infected mice. C57BL6 mice or BALB/c mice were infected in the footpads and sacrificed after 10 weeks. Lymphocytes (dLNs) were isolated and plated on 24-well dishes. After SLA stimulation for 3 days, culture supernatants were collected to measure the level of IFN-γ (**A**), IL-4 (**B**), and IL-10 (**C**). Ratios of SLA-stimulated/un-stimulated were calculated for each cytokine (**A**–**C**). Ratios of IL-4/IFN-γ and IL-10/IFN-γ (both from SLA-treated samples) were also calculated (**D**–**E**). Error bars represent standard deviations from 3 replicates (*: *p*<0.05).

The reduced virulence of *Laiscl* ^−^ parasites in C57BL6 mice but not in BALB/c mice prompted us to examine whether these mutants elicited a different cytokine response from *La* WT parasites. At 10 weeks post infection, the dLN cells of infected mice were stimulated with SLA and the production of IFN-γ, IL-4 and IL-10 were measured as previously described [9]. In C57BL6 mice, *L. amazonensis* infection induced high levels of IFN-γ (Fig. 7A). Comparing to *La* WT and *Laiscl* ^−^ */+LaISCL*, *Laiscl* ^−^ parasites triggered more of IL-4 and IL-10 production (the left side of Fig. 7B–C), although only the difference in IL-4 level is statistically significant. In BALB/c mice, *L. amazonensis* infection led to over production of IL-4 and IL-10 but very low levels of IFN-γ, while no statistically significant difference was detected between *Laiscl* ^−^ and *La* WT or *Laiscl* ^−^/*+LaISCL* parasites (the right side of Fig. 7A–C). As indicated by the ratios of IL-4/IFN-γ and IL-10/IFN-γ, *L. amazonensis* parasites induced a Th1-biased response in C57BL6 mice and a Th2-biased response in BALB/c mice (Fig. 7D–E). It is of note that these biases are not as extreme as those with *L. major* infection, which is strictly Th1-dominated in C57BL6 mice and Th2-dominated in BALB/c mice [9], [17]. In summary, loss of SL degradation in *L. amazonensis* did not significantly alter the cytokine production in BALB/c mice, but it did result in a slight increase in IL-4 and IL-10 expression in C57BL6 mice.

## Discussion

SL degradation plays multiple roles in *L. major*: while the IPCase activity is important for promastigote survival and acid tolerance, the SMase activity is required for amastigote proliferation in mice and the manifestation of disease [9]. In this study, we investigated whether the function of SL degradation is conserved in another *Leishmania* species, *L. amazonensis*. Unlike *L. major*, *L. amazonensis* parasites cause non-healing lesions in almost all inbred strains of mice in the absence of a Th2 dominance [18] [19].

Biochemically, LaISCL resembles *L. major* ISCL in that it is responsible for the hydrolysis of both IPC and sphingomyelin (Fig. 5). LaISCL protein is highly expressed in the infective stages and is strongly associated with the mitochondria (Figs. 3, 4). For promastigotes, losing LaISCL leads to hypersensitivity to acidic pH and poor viability in late stationary phase (Figs. 2 and S2). As axenic amastigotes, *Laiscl^−^* mutants exhibit a slower growth rate and reduced survival than *La* WT (Fig. 2). Importantly, both promastigotes and lesion-derived amastigotes of *Laiscl^−^* were fully infective towards BALB/c mice yet showed severely attenuated virulence towards C57BL6 mice (Fig. 6). This phenotype makes the *Laiscl^−^* mutants somewhat similar to the *L. major* wild type parasites but drastically different from the *L. major iscl^−^* mutants which are completely avirulent in both BALB/c and C57BL6 mice [9].

While the degradation of sphingomyelin or IPC is dispensable for *L. amazonensis* to establish infection in BALB/c mice, *Laiscl^−^* mutants do not survive well in BALB/c macrophages *in vitro* and this defect is clearly due to the loss of LaISCL (Fig. S6). Potentially, in BALB/c mice, other host factors such as cytokines, neutrophils, dendritic cells, or T cells may interact with macrophages in a way that benefits the survival and proliferation of *Laiscl^−^*. It is not clear why the outcome of *Laiscl^−^* infection is dependent on mouse genetic background. Among common inbred mouse strains, there is widespread variation in the number and function of natural killer T cells (NKT-cells) [38]. These cells can recognize and be activated by the CD1d-presented glycosphingophospholipid antigen from *Leishmania* to promote parasite killing [39], [40]. The SL degradation defect in *Laiscl^−^* could alter the production and/or presentation of glycosphingophospholipid antigen, and thus affects its interaction with NKT cells. Compared to BALB/c mice, C57BL6 mice contain a higher number of NKT cells [38] which may lead to a more effective control of *Laiscl^−^* parasites.

As shown in Fig. 7A–C, BALB/c mice infected by *Laiscl^−^* produced similar levels of IFN-γ, IL-4, and IL-10 as those infected by *La* WT or *Laiscl^−^*/+ *LaISCL* parasites. In C57BL6 mice, however, *Laiscl^−^* triggered significantly more IL-4 production than *La* WT or *Laiscl^−^*/+ *LaISCL* parasites (Fig. 7B). While IL-4 is primarily a susceptibility factor for *L. amazonensis* infection in BALB/c mice [18], [41], its role in C57BL6 or C3H mice is less clear [18], [42]. A previous study indicates that the level of IL-4 correlates with lesion development in *L. amazonensis*-infected C57BL10 mice [18]. Therefore, the increased IL-4 production from *Laiscl^−^*-infection may be the consequence, rather than the cause of reduced virulence (Figs. 6, 7).

Clearly, *L. amazonensis* infection of C57BL6 mice led to significant IFN-γ production (Fig. 7A). Effect of IFN-γ on *L. amazonensis* infection can be complex. On one hand, this cytokine can activate murine macrophages and inhibit parasite growth when it is applied in combination with LPS [21], [43]. On the other hand, without LPS, IFN-γ alone can improve parasite invasion and replication in macrophages [43], [44]. Mechanism of such an infection-promoting effect is not well defined, although the induction of autophagy via IFN-γ treatment could be involved [45]. Loss of LaISCL did not affect the production of IFN-γ in C57BL6 mice but did limit the replication of *Laiscl^−^* (Figs. 6, 7). This result suggests that SL degradation may be involved in balancing the dual effects of IFN-γ, which is crucial for *L. amazonensis* proliferation in C57BL6 mice.

In summary, we demonstrate that the role of SL degradation in *Leishmania* virulence can vary significantly among different parasite species and is highly dependent on the mammalian host. Similar to murine infections, the genetic background of human host also has a major influence in the outcome of *Leishmania* infection [46]–[48]. To further evaluate the potential of SL degradation as a drug target, future studies may expand the investigation to other *Leishmania* species and other hosts. Another potential point of interest from our study is that the *Laiscl^−^* mutants resemble wild type *L. major* parasites in mouse infections (fully virulent in BALB/c mice but severely attenuated in C57BL6 mice). Since *L. amazonensis*–infection can cause a wider range of symptoms in humans than *L. major*–infection, it would be interesting to examine whether LaISCL is required for the dissemination of *L. amazonensis* and the manifestation of DCL, a rare but difficult disease to treat.

## Supporting Information

Figure S1 **Sequence alignment of** ***L. amazonensis*** **ISCL (LaISCL) and** ***L. major*** **ISCL (LmISCL).** Alignment was done using the NCBI BLASTp program. Non-identical amino acids are shown in red. The underlined sequence indicates the P-loop motif. Amino acids 241–256 of LmISCL (boxed area) represent the epitope recognized by the anti-LmISCL peptide antibody. The braces represent predicted transmembrane helices. Asterisks mark amino acids that are essential for catalysis based on a recent study of LmISCL [10].
(PDF)

Figure S2 **Ability of** ***Laiscl*** **^−^ mutants to survive under acidic conditions.** Promastigotes were cultured to stationary phase in either regular media (pH 7.4, **A–B**) or acidic media (pH 5.0, **C–D**). Cell density (**A** and **C**) and viability (**B** and **D**) were measured daily after entry into stationary phase. Black bars: *La* WT; white bars: *Laiscl^−^*; grey bars: *Laiscl^−^/+LaISCL*. Experiments were repeated three times and error bars represent standard deviations (*: *p*<0.05, **: *p*<0.01).
(PDF)

Figure S3 **Metacyclogenesis is normal in** ***Laiscl^−^*** **mutants.** *La*WT (•), *Laiscl^−^* (○) and *Laiscl^−^/+LaISCL* (▾) promastigotes (*in vitro* passage numbers <5) were cultured to stationary phase. Metacyclics were purified using the density centrifugation method [27] and percentages of metacyclics were determined daily.
(PDF)

Figure S4 **Localization of LaISCL in amastigotes.** *La* WT amastigotes were isolated from infected BALB/c mice and analyzed by immunofluorescence microscopy. (**A**) phase contrast image; (**B**) DNA staining using Hoechst 33242; (**C**) labeling with Mitotracker Red 580; (**D**) immuno-staining with rabbit anti-LmISCL antibody, followed by FITC conjugated goat-anti-rabbit IgG; (**E**) merge of **C** and **D**.
(PDF)

Figure S5 ***Laiscl*** **^−^ mutants contain an increased level of IPC.** Total lipids were extracted from stationary phase promastigotes of *La* WT (**A**), *Laiscl* ^−^ (**B**), or *Laiscl^−^/+LaISCL* (**C**) and analyzed by electrospray ionization mass spectrometry as previously described [31]. Representative spectra (negative ion mode) are shown with major phospholipids labeled (IPC: inositol phosphorylceramide, PE: phosphatidylethanolamine, PI: phosphatidylinositol).
(PDF)

Figure S6 ***Laiscl*** **^−^ parasites survive poorly in murine macrophages (MÖs).** Bone marrow MÖs from BALB/c mice (**A**–**B**) or C57BL6 mice (**C**–**D**) were infected by stationary phase promastigotes of *La* WT(•), *Laiscl* ^−^ (○), or *Laiscl* ^−^/+*LaISCL* (▾). As a control, *La* WT parasites were also used to infect MΦs that were activated with 50 ng/ml of LPS and 50 ng/ml of IFN-γ (▵). Fraction of infected MΦs (**A**, **C**) and number of parasites per 100 MΦs (**B**, **D**) were recorded. Error bars represent standard deviations.
(PDF)

Table S1 **List of oligonucleotides.** Sequences shown in lowercase represent restriction enzyme sites.
(PDF)
