# Supplementary material for: Sphingolipid Degradation in Leishmania (Leishmania) amazonensis
Source: PLoS Negl Trop Dis. 2012 Dec 20;6(12):e1944. doi: 10.1371/journal.pntd.0001944 (PMC3527339; doi:10.1371/journal.pntd.0001944)
Supplement: Figure S1 — Sequence alignment of L. amazonensis ISCL (LaISCL) and L. major ISCL (LmISCL). Alignment was done using the NCBI BLASTp program. Non-identical amino acids are shown in red. The underlined sequence indicates the P-loop motif. Amino acids 241–256 of LmISCL (boxed area) represent the epitope recognized by the anti-LmISCL peptide antibody. The braces represent predicted transmembrane helices. Asterisks mark amino acids that are essential for catalysis based on a recent study of LmISCL [10]. (PDF) [file pntd.0001944.s001.pdf]

```
LaISCL 1    MSHVSTFAADELPIRVLSFNLWGIFNSKMREARMKAFATKIEHYDVILLQEQFSAEDFDL
LmISCL 1    MSHASTFAAGELPIRVLTFNLWGIFNSRMREARMKVFATKIEHYDVILLQEQFSVEDFDL

                                                                    *
LaISCL 61   IFQHASPVVQRTYTFRRFCSSFYGSGCAVISRYPICQAFFHTFPLQGCPEMVLHGDFFAN
LmISCL 61   IFQNASPVVQRTYTFRRFCSSFYGSGCAVISRYPISQAFFHTFPLQGYPEMVLHGDFFAN

LaISCL 121  KGAAMVRVMVPVTMKDGSAAKAQ-VTLYTTHLVAVYEKVSQLSSWKRERYLPFRISQAIS
LmISCL 121  KGAAMVRVMVPVTMADGGAAKAQEVTLYTTHLVAVYEKVSQLSSWRRERYLPFRISQAIS

                                  *
LaISCL 180  FADFIVSTSRPTDRIIIGGDFNCSQRSLEVQMMLILLKRCGYNMHSVLPPPRALRDAATV
LmISCL 181  FADFIVSTSRPTDPIIIGGDFNCSQRSLEVQMMLILLKRYGYDMHSVLPPPRALRDAAAA

LaISCL 240  GQECDDVQRFFTYSDRNAFNSMKTSYFKLLKLEADIPSQIDHIFFSRPAFALRQFADCPD
LmISCL 241  EQEREVGQRFFTYSDRNTFNSMKTSYFKLLKLEADIPSQIDHMFFSRPAFALLQFADCPD

LaISCL 300  VAEGYPCTLRDAPNGLVVFTKSEVYVPPHSTWYGSLWHQLLTAKRMPRGANATGQLAKSG
LmISCL 301  VADGYPCVLQDAPNGLVVFTKNEVHVPPHSAWYGSLWHQLFSGKRVPRGANATGQLAKLR

                                  *
LaISCL 360  SKTALSTEEQSADDAAHYYPISDHFGVAALLGMRVETVG-------SSAETGDAAALALT
LmISCL 361  CKAASSTEGQSADDAAHYYPMSDHFGVAALLGMRVEKVDSTTAMICSSAGTGGTAALALT

LaISCL 413  PDEARAVQTVADFLEDYVCKLRSQAKTTRYMAVLSVLLVAANIWVLRQLSAKEEVRSAAV
LmISCL 421  PEEARAVQTVVAFLEDYVRKLRSQAKTARYMAVFSLLLVATNIWVLRRLSAKEEARSAAV

LaISCL 473  LEHLYDMAAATTRDTAKVVQQDKGLESIKHGFNVAKDWVSNQAHLTLRIVNKVTGA----
LmISCL 481  LERIYDMAAAATRDTAMVVQPGKGLESIKHGFNTAKDWVNNQAHLTLHIVSKFTGGTPAP

LaISCL 529  GIDACDTPEGSKQPAANVADPSEPVPRTVPASTAARTAKRATTTLEGDAAAFAAVRPDFR
LmISCL 541  GINACDTPEGSKQPAANVADPSEPVPRTVPASTTATTAKRATTTPEGDAAA----RPDFR

LaISCL 589  AIAEALTARPLYASTWVSSAFNMTAAVVGTVSFAIGVFQRAGNANILEEQVHQLKKL
LmISCL 597  AIAEALTVRPLYASAWVSSAFNITAAVVGTVSFAIGVFQRAGNANVLEEQAHQLKKL
```
