# Supplementary figures and images for: Sphingolipid Degradation in Leishmania (Leishmania) amazonensis
Source: PLoS Negl Trop Dis. 2012 Dec 20;6(12):e1944. doi: 10.1371/journal.pntd.0001944 (PMC3527339; doi:10.1371/journal.pntd.0001944)

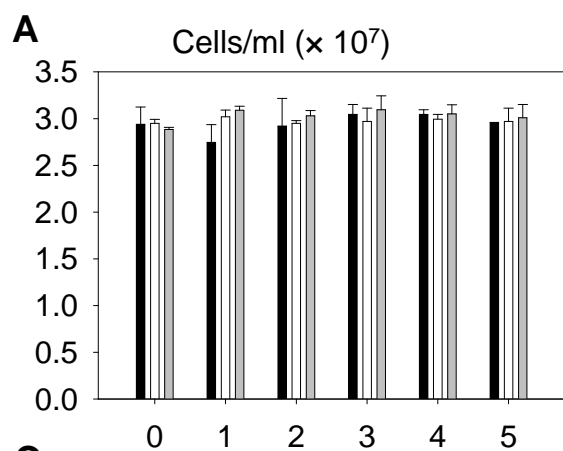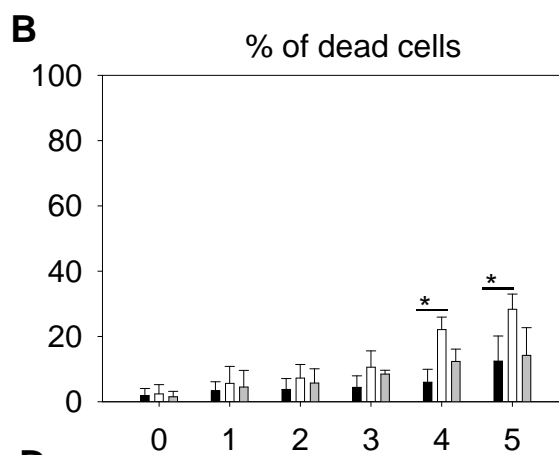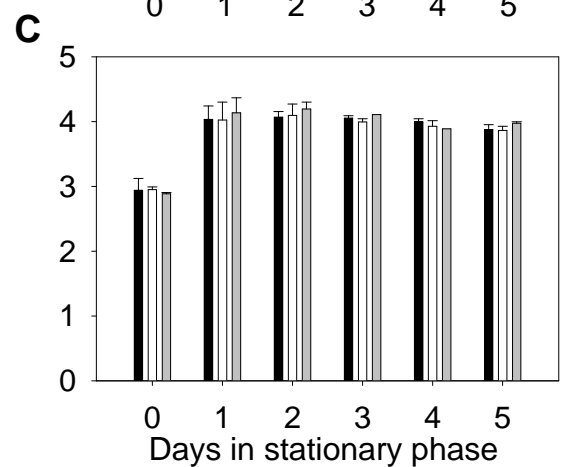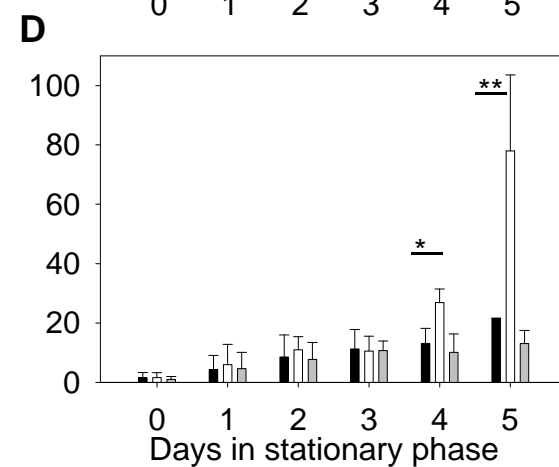

Supplement: Figure S2 — Ability of Laiscl − mutants to survive under acidic conditions. Promastigotes were cultured to stationary phase in either regular media (pH 7.4, A–B) or acidic media (pH 5.0, C–D). Cell density (A and C) and viability (B and D) were measured daily after entry into stationary phase. Black bars: La WT; white bars: Laiscl−; grey bars: Laiscl−/+LaISCL. Experiments were repeated three times and error bars represent standard deviations (*: p<0.05, **: p<0.01). (PDF) [file pntd.0001944.s002.pdf]

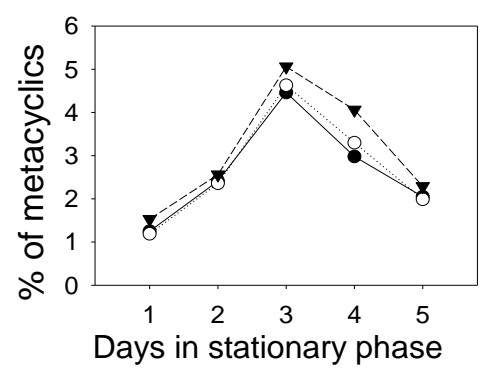

Supplement: Figure S3 — Metacyclogenesis is normal in Laiscl− mutants. LaWT (•), Laiscl− (○) and Laiscl−/+LaISCL (▾) promastigotes (in vitro passage numbers <5) were cultured to stationary phase. Metacyclics were purified using the density centrifugation method [27] and percentages of metacyclics were determined daily. (PDF) [file pntd.0001944.s003.pdf]

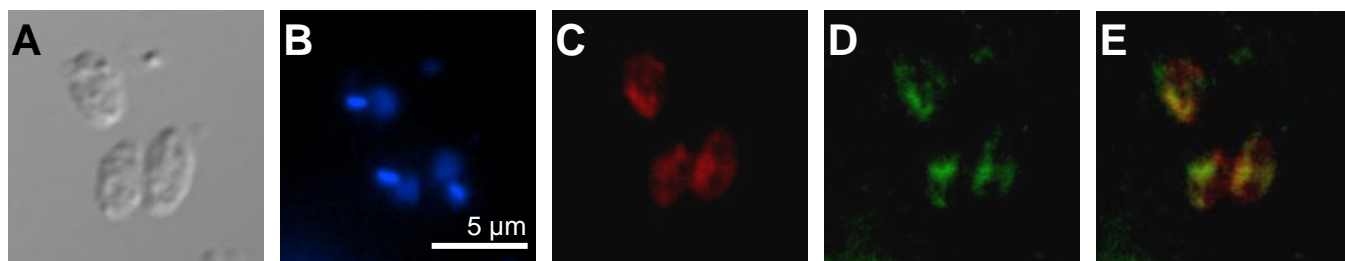

Supplement: Figure S4 — Localization of LaISCL in amastigotes. La WT amastigotes were isolated from infected BALB/c mice and analyzed by immunofluorescence microscopy. (A) phase contrast image; (B) DNA staining using Hoechst 33242; (C) labeling with Mitotracker Red 580; (D) immuno-staining with rabbit anti-LmISCL antibody, followed by FITC conjugated goat-anti-rabbit IgG; (E) merge of C and D. (PDF) [file pntd.0001944.s004.pdf]

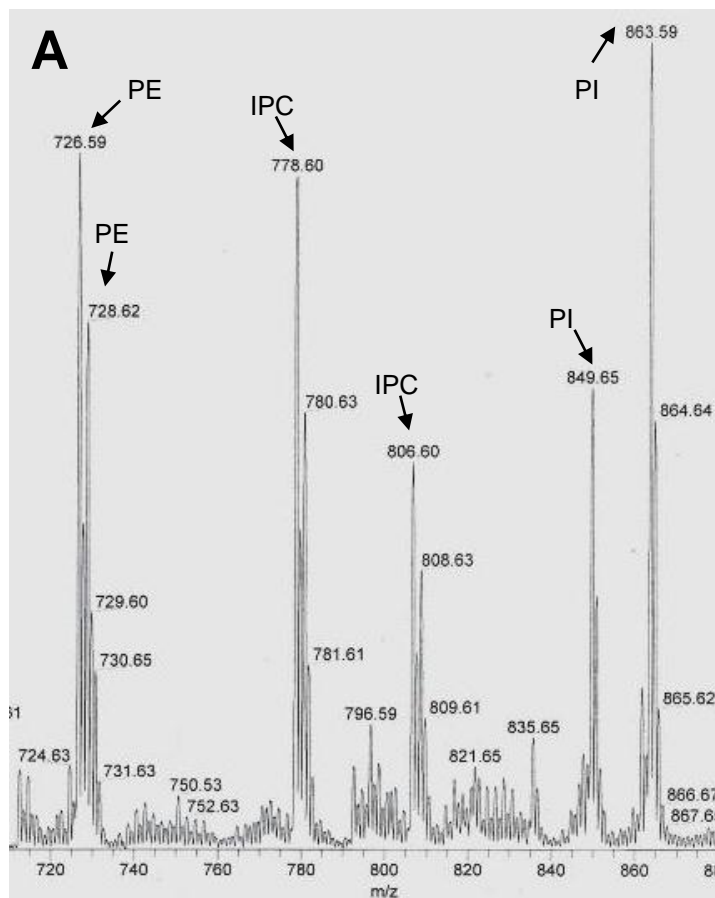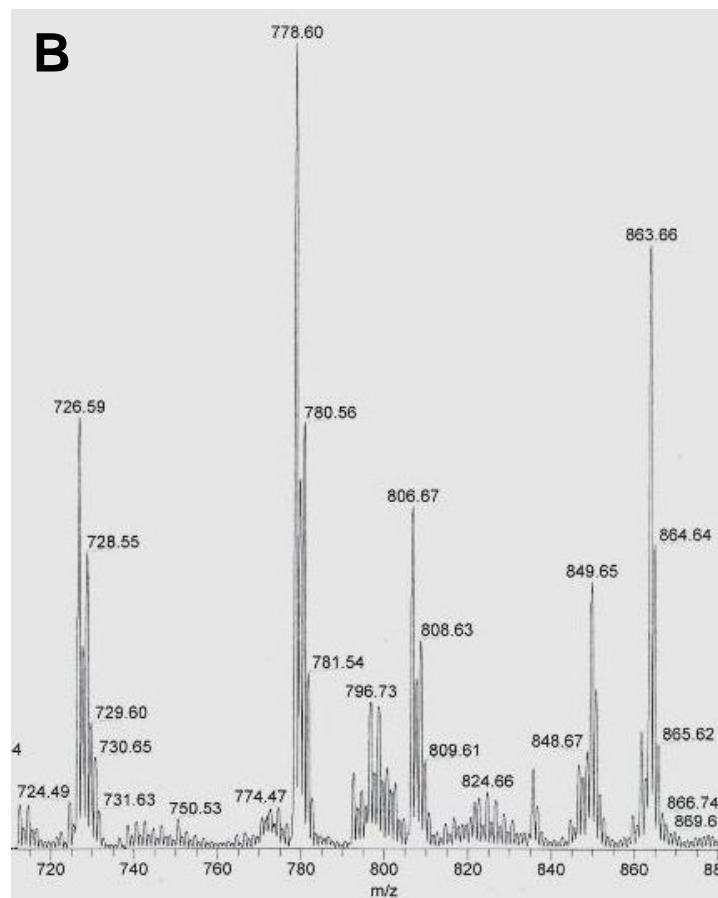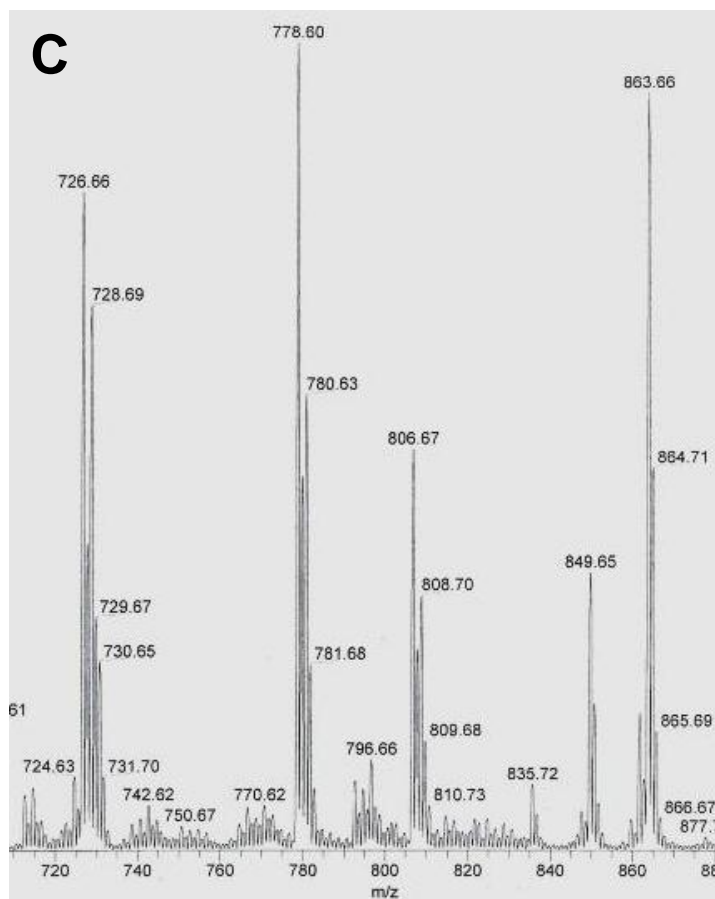

Supplement: Figure S5 — Laiscl − mutants contain an increased level of IPC. Total lipids were extracted from stationary phase promastigotes of La WT (A), Laiscl − (B), or Laiscl−/+LaISCL (C) and analyzed by electrospray ionization mass spectrometry as previously described [31]. Representative spectra (negative ion mode) are shown with major phospholipids labeled (IPC: inositol phosphorylceramide, PE: phosphatidylethanolamine, PI: phosphatidylinositol). (PDF) [file pntd.0001944.s005.pdf]

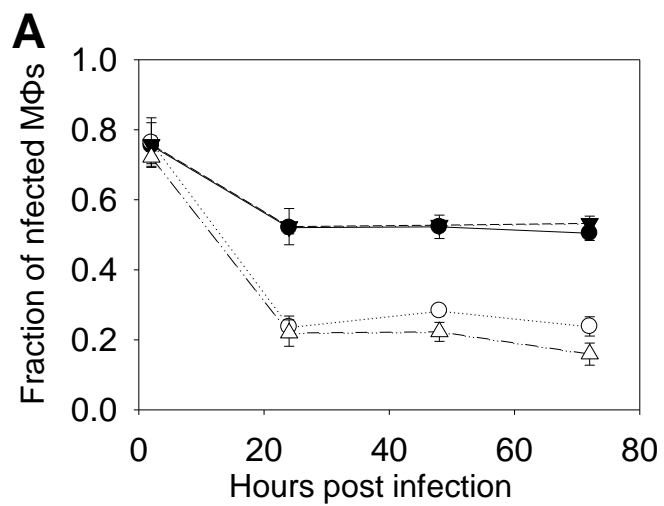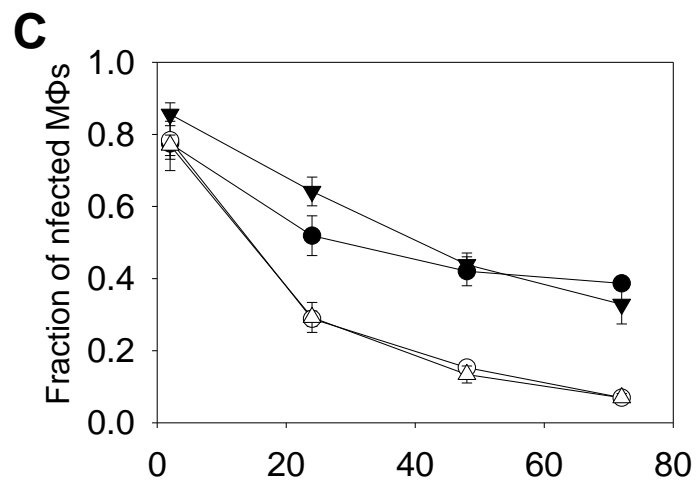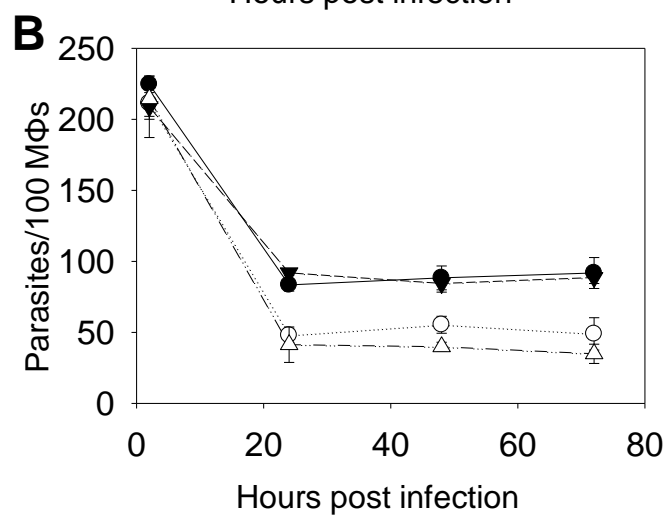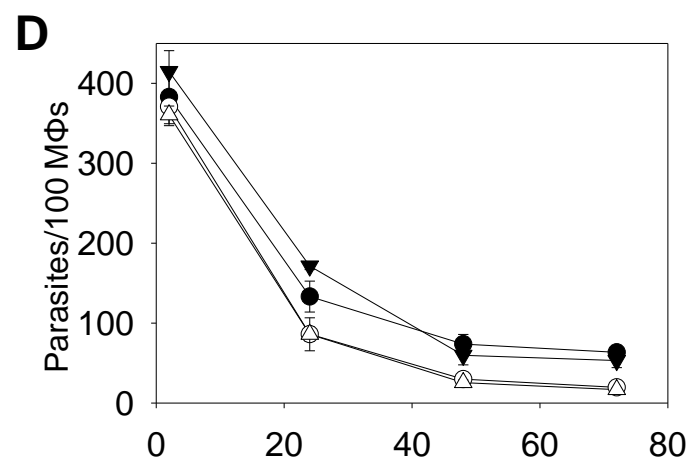

Supplement: Figure S6 — Laiscl − parasites survive poorly in murine macrophages (MÖs). Bone marrow MÖs from BALB/c mice (A–B) or C57BL6 mice (C–D) were infected by stationary phase promastigotes of La WT(•), Laiscl − (○), or Laiscl −/+LaISCL (▾). As a control, La WT parasites were also used to infect MΦs that were activated with 50 ng/ml of LPS and 50 ng/ml of IFN-γ (▵). Fraction of infected MΦs (A, C) and number of parasites per 100 MΦs (B, D) were recorded. Error bars represent standard deviations. (PDF) [file pntd.0001944.s006.pdf]
