# Supplementary material for: Sphingolipid Degradation in Leishmania (Leishmania) amazonensis
Source: PLoS Negl Trop Dis. 2012 Dec 20;6(12):e1944. doi: 10.1371/journal.pntd.0001944 (PMC3527339; doi:10.1371/journal.pntd.0001944)
Supplement: Table S1 — List of oligonucleotides. Sequences shown in lowercase represent restriction enzyme sites. (PDF) [file pntd.0001944.s007.pdf]

Primer #	Name	Sequence
141	5’ LaISCL ORF (BamHI)	ATTACTggatccACCATGTCGCACGTGTCGACCTT
59	3’ LmISCL ORF (BamHI)	ATTACTggatccCTACAACTTCTTCAGCT
161	Forward 5‘ flanking LaISCL (EcoRI)	TCCTCCTgaattcCACGCACACGCAACACTCGC
143	Reverse 5’ flanking LaISCL (SpeI + BamHI)	TGCTGTggatccAGTTGGactagtGTTGTCCAGGTCTCCCTCTCCCTC
144	Forward 3’ flanking LaISCL (BamHI)	ACATCAggatccTGTGCACTGTGCGTGCGCC
145	Reverse 3’ flanking LaISCL (HindIII)	TGATGTaagcttGCGTTAGGGGTCTACTCAACC
